# Vitamin D receptor and megalin gene polymorphisms are associated with central adiposity status and changes among US adults

**DOI:** 10.1017/jns.2013.19

**Published:** 2013-10-30

**Authors:** May A. Beydoun, Toshiko Tanaka, Hind A. Beydoun, Eric L. Ding, Luigi Ferrucci, Alan B. Zonderman

**Affiliations:** 1 National Institute on Aging, NIA/NIH/IRP, Baltimore, MD, USA; 2 Graduate Program in Public Health, Eastern Virginia Medical School, Norfolk, VA, USA; 3 Harvard School of Public Health, Department of Nutrtion, Boston, MA, USA

**Keywords:** Central adiposity, SNP, Vitamin D receptor, Megalin, Adults, BLSA, Baltimore Longitudinal Study of Aging, ECA, elevated central adiposity, LCA, latent class analysis, LD, linkage disequilibrium, SICA, significant increase in central adiposity, SNPHAP, SNP halotype, SNPLC, SNP latent class, VDR, vitamin D receptor, WC, waist circumference, WHR, waist:hip ratio

## Abstract

We examined longitudinal associations of vitamin D receptor (VDR) and megalin (LRP2; LDL receptor-related protein-2) gene polymorphisms with central adiposity. We used data from the Baltimore Longitudinal Study of Aging (BLSA), an ongoing prospective open cohort study. Study participants consisted of non-Hispanic white adults residing in Baltimore city, with one or more visits at age ≥50 years, and complete data (*n* 609–617). Repeated assessments on waist circumference (WC) and waist:hip ratio (WHR) were available. Multiple linear mixed models were used to estimate mid-follow-up age central adiposity level and annual rate of change with cut-points set at the sex-specific 80th percentile. The four binary outcomes were: ‘elevated central adiposity’ (ECA-WC and ECA-WHR) and ‘significant increase in central adiposity’ (SICA-WC and SICA-WHR). SNP for VDR (four SNP: (1) rs11568820 (CdX-2:T/C); (2) rs1544410 (BsmI:G/A); (3) rs7975232 (ApaI:A/C); (4) rs731236 (TaqI:G/A)) and Megalin (three SNP: (1) rs3755166:G/A; (2) rs2075252:C/T; (3) rs4668123:C/T) genes were selected. SNP latent classes (SNPLC) and SNP haplotypes (SNPHAP) were created. Multiple logistic regression analyses indicated that, in men, higher ECA-WHR odds were associated with SNPLC Megalin_2_:rs3755166[–]/rs2075252[TT]/rs4668123[T–] (*v*. Megalin_1_:rs3755166[–]/rs2075252[CC]/rs4668123[–]) (OR 2·87; 95 % CI 1·15, 7·12; *P* = 0·023) and that SNPLC Megalin_3_:rs3755166[–]/rs2075252[CT]/rs4668123[–] (*v*. Megalin_1_) was linked to lower SICA-WC odds (OR 0·48; 95 % CI 0·26, 0·88; *P* = 0·019) (*P* > 0·05 for sex × SNPLC). In women, VDR_3_ SNPHAP (GAA:bAT) was related to lower odds of ECA-WC (OR 0·37; 95 % CI 0·16, 0·87; *P* = 0·023) (*P* < 0·05 for sex × SNPHAP), VDR_1_ SNPHAP (GCA:baT) was associated with greater odds and VDR_3_ SNPHAP (GAA:bAT) with lower odds of SICA-WC (*P* > 0·05 for sex × SNPHAP). Vitamin D-related gene polymorphisms were associated with central adiposity status and change. Future mechanistic studies are needed to confirm those polymorphisms' biological significance to central adiposity.

Human adiposity is heritable and polygenic^(^^1^^)^, with genes contributing 16–85 % for BMI^(^^2^^)^ and 37–81 % for waist circumference (WC) (for example, Hunt *et al.*^(^^3^^)^). Gene–environment interactions may largely determine adiposity phenotypes. Moreover, serum 25-hydroxyvitamin D (25(OH)D) concentration correlated inversely with adiposity and related metabolic disorders^(^^4^^)^. The lower bioavailability of the fat-soluble vitamin D through its sequestration into excessive fat tissues was a suggested mechanism^(^^5^^)^, implicating obesity in the aetiology of vitamin D deficiency. Conversely, vitamin D may play a causal role in obesity by modulating homeostasis of intracellular Ca which correlates inversely with dairy product consumption. Ultimately, a higher intracellular Ca triggers lipogenesis and suppresses lipolysis^(^^6^^)^.

Vitamin D's active form (1,25-dihydroxyvitmain D_3_ (1,25(OH)_2_D_3_)) binds directly to the nuclear vitamin D receptor (VDR). The VDR gene is located on chromosome 12 and contains fourteen exons (chr12q13·1). The VDR–1,25(OH)_2_D_3_ complex modulates transcription of vitamin D-responsive genes^(^^7^^)^ and influences adipocyte differentiation both *in vitro* and *in vivo*^(^^8^^)^. Epidemiological studies show associations of VDR gene polymorphisms with adiposity and related metabolic disorders^(^^9^^–^^17^^)^. However, most studies specifically examining adiposity outcomes had small sample sizes (<400) (for example, Grundberg *et al.*^(^^10^^)^, Filus *et al.*^(^^11^^)^ and Speer *et al.*^(^^15^^)^), some were restricted to one sex (for example, Ochs-Balcom *et al.*^(^^9^^)^ and Grundberg *et al.*^(^^10^^)^) but, more importantly, all were cross-sectional or case–control by design^(^^9^^–^^17^^)^. Thus, our present study is, to our knowledge, the first one to examine VDR gene polymorphisms in relation to longitudinal adiposity outcomes.

Another endocytic vitamin D-binding receptor, known as megalin (or LDL receptor-related protein-2; LRP2), is expressed in many epithelial cells, belongs to the LDL receptor family and its expression is directly regulated by both vitamin D and vitamin A^(^^18^^)^. Vitamin D enters cells via megalin receptor bound to vitamin D-binding protein^(^^19^^)^. Megalin influences obesity possibly by mediating the transport of the appetite-regulating adipokine leptin through the blood–brain barrier and modulates leptin signaling^(^^20^^)^. Leptin, in turn, was linked to vitamin D metabolism by attenuating gene expression of renal enzyme 25-hydroxyvitamin D_3_-1α-hydroxylase in mice^(^^21^^)^. Megalin also facilitates transcytosis of precursor hormone thyroglobulin^(^^22^^)^. Leptin and thyroid hormone collectively affect adiposity through their regulation of energy metabolism, thermogenesis, glucose and lipid metabolism, appetite and food intake, and the oxidation of fatty acids^(^^23^^)^. Megalin is also a receptor for sex hormone-binding globulin. In fact, a cross-effect modification of oestrogen and vitamin D interventions was observed for colorectal cancer incidence in the Women's Health Initiative trial^(^^24^^)^, suggesting an interplay of oestrogen and vitamin D via megalin and a possible differential effect of megalin polymorphisms between sexes. To our knowledge, aside from recent genome-wide association studies (for example, Heid *et al.*^(^^25^^)^), no study using a candidate gene approach has thus far examined megalin gene polymorphism in relation to adiposity phenotypes, particularly longitudinal changes in central adiposity, though these variations were tested for outcomes such as cognition and dementia^(^^26^^)^, a phenotype shown to be associated with obesity in a recent meta-analysis^(^^27^^)^.

In our present study, we hypothesise that selected polymorphisms in VDR and megalin genes previously shown to affect various metabolic and cardiovascular health outcomes, mainly in cross-sectional studies, are also associated with central adiposity status and longitudinal changes in a sample of non-Hispanic white US adults.

## Materials and methods

### Database and study subjects

Data from the Baltimore Longitudinal Study of Aging (BLSA) were used, with methods summarised elsewhere^(^^28^^)^. Eligible participants for our present study had at least one visit at or beyond age 50 years (*n*_2_ 2321 of 3005), and were restricted further to non-Hispanic whites (*n*_3_ 1917), given possible differential associations of vitamin D status with adiposity in different ethnic groups. Complete genetic data among those non-Hispanic white participants eligible for analysis were available for *n*_4_ 702 BLSA participants, of whom *n*_4_ 609–617 had complete central adiposity and covariate measurements. The present study was conducted according to the guidelines laid down in the Declaration of Helsinki and all procedures involving human subjects were approved by the the Institutional Review Board (IRB) of Medstar Health Research Institute. Written informed consent was obtained from all subjects. In addition, genetic and other variables were de-identified for the purpose of statistical analysis.

### Data collection and key measurements

#### Classification of elevated central adiposity and significant increase in central adiposity

BLSA staff clinicians assessed WC with a tape measure kept parallel to the floor, from the hipbone and wrapping around the waist at navel level while participants were holding their breath. Hip circumference was similarly measured and waist:hip ratio (WHR) was computed accordingly. Multiple assessments were available (WC, *n* 14 852 visits, *n* 2886 subjects; WHR, *n* 14 832 visits, *n* 2886 subjects) and mean and range of individual assessments (or visits) were: WC, mean 5·1 (range 1–25); WHR, mean 5·1 (range 1–25, with more than 90 % of participants having at least two visits.

We conducted linear mixed models to predict individual WC and WHR at mean follow-up age and estimate annual rate of change between age 50 years and mean follow-up age (see online Supplementary Material S2), an approach previously used to predict cognitive performance and annual rate of change^(^^26^^)^. Using sex-specific quintiles, binary outcomes ‘elevated central adiposity’ (ECA) and ‘significant increase in central adiposity’ (SICA) were defined as the uppermost quintile (value = 1) for central adiposity level and annual rate of change, respectively, and compared with all other quintiles combined (value = 0). ECA and SICA were defined for WC and WHR, and thus four binary outcomes were obtained (ECA-WC, ECA-WHR, SICA-WC and SICA-WHR). The choice of the binary outcome (as opposed to a continuous one) was driven by the potential clinical significance of the effects as well as the ease of interpretation and replication in future studies that would use similar cut-points in independent samples.

#### Genotyping strategy and gene polymorphism classification: SNP, SNP latent classes and SNP haplotypes

DNA, extracted from collected blood samples, was used for genome-wide genotyping on 1231 BLSA participants with Illumina 550K. HapMap-CEU (http://hapmap.ncbi.nlm.nih.gov/; build 36) was also used to impute approximately 2·5 million SNP with MACH^(^^29^^)^. CEU is a population sample of Utah residents with Northern and Western European ancestry from the CEPH (Council on Education for Public Health) collection. SNP with imputation quality *r*^2^ < 0·3 or minor allele frequency of <1 % were excluded. SNP were selected from findings of confirmatory candidate gene studies of adiposity or various health outcomes that are linked to adiposity^(^^9^^–^^16^^,^^26^^)^. Most VDR SNP were available in our database, with few exceptions (for example, VDR SNP rs10735810, *Fok*I:G/A). Consequently, four VDR SNP (rs11568820 (*Cdx*-2:T/C); rs1544410 (*Bsm*I:G/A); rs7975232 (*Apa*I:A/C); rs731236 (*Taq*I:G/A)) and three megalin SNP (rs3755166:G/A; rs2075252:C/T; rs4668123:C/T) were chosen (online Supplementary material S1, Fig. S1(a) and Fig. S1(b)).

Using latent class analysis (LCA) with sex and first-visit age as covariates and selected SNP entered into that model (one gene per model) as a three-level categorical variable, VDR and megalin SNP latent classes (SNPLC) were obtained (PROC LCA in SAS version 9.1; SAS Institute Inc.)^(^^30^^)^. Akaike information and Bayesian information criteria for model fit determined the appropriate number of latent classes. Using the Bayes theorem, posterior probabilities were estimated and were identical for all individuals with a particular SNP pattern per gene. Each individual belonged to a specific SNPLC when the posterior probability for this class was >0·50. For most individuals, the expected posterior probability is >0·90 for a specific latent class^(^^30^^)^ (online Supplementary material S1, Fig. S1(a) and Fig. S1(b)).

Additionally, using Haploview version 4.2^(^^31^^)^, SNP haplotypes (SNPHAP) per gene were also created. For the VDR gene, three SNP (*Bsm*I, *Apa*I and *Taq*I) with moderately strong linkage disequilibrium (LD) were combined into SNPHAP, as was done in previous studies (for example, Beydoun *et al.*^(^^26^^)^). Consequently, three SNPHAP were prevalent in our population, combining *Bsm*I, *Apa*I and *Taq*I as follows: VDR_1_, GCA (baT), VDR_2_, AAG (BAt) or VDR_3_, GAA (bAT) for one or two alleles. We coded participants as: 0 = having no VDR_x_ haplotype; 1 = having one allele carrying the VDR_x_ haplotype; 2 = having two alleles carrying the VDR_x_ haplotype. Using a similar approach, eight megalin haplotypes were uncovered. However, only three megalin SNPHAP were selected for our analysis (prevalence with one or two copies was >10 %). Both SNPLC and SNPHAP have been created and used in a similar fashion in a previous study^(^^26^^)^.

#### Other main covariates

To adjust for potential confounding in the main associations of interest, three sets of covariates were considered: (1) sociodemographic factors, namely first-visit age and mean follow-up ages (per individual and outcome), sex, educational attainment (years of schooling), and one lifestyle-related factor, namely smoking status (never, former or current smoker); (2) self-reported history of type 2 diabetes, hypertension, CVD (stroke, congestive heart failure, non-fatal myocardial infarction or atrial fibrillation) and dyslipidaemia at first visit. Only covariates (1) were considered as potential confounders in multiple regression models, whereas (2) were only used for descriptive purposes. In addition, EIGENSTRAT analysis (implemented as part of the EIGENSOFT package) was conducted and two top principal components were added in multiple regression models to control for any residual effects of population structure as described in a previous study^(^^26^^)^. The EIGENSTRAT method is a program that conducts principal components analysis to correct for stratification in genome-wide association studies.

### Statistical analysis

For each selected SNP, we assessed Hardy–Weinberg equilibrium with an exact test, using Haploview version 4.2^(^^31^^)^, and calculated pair-wise LD. Online Supplementary Material S3 shows the LD map for all available VDR and megalin SNP. We present means and proportions of study sample characteristics, gene SNP, SNPLC and SNPHAP distributions.

Further, we conducted multiple logistic regression analyses to examine associations of VDR and megalin SNP, SNPLC and SNPHAP with four binary central adiposity: (1) ECA-WC; (2) ECA-WHR; (3) SICA-WC; (4) SICA-WHR (see online Supplementary Material S2).

A two-stage Heckman selection model was constructed to account for selection bias due to non-random participant selection for genetic analysis, as was done in several other studies (for example, Beydoun *et al.*^(^^26^^)^). At a first stage, a probit model produced an inverse Mills ratio (IMR), directly derived from the predicted probability of being selected, conditional on model covariates. To adjust for this selection bias, the IMR entered the main multiple logistic regression models as a covariate in the second stage. Stratified analyses by sex were conducted. Effect modification by sex was tested by adding interaction terms of sex with SNP, SNPLC and SNPHAP. In particular, sex differences in the association between megalin gene polymorphism and various phenotypes including adiposity were hypothesised *a priori*^(^^24^^)^.

With the null hypothesis being no association between SNP (or SNPHAP, SNPLC) and the four main outcomes of interest ((1) ECA-WC; (2) ECA-WHR; (3) SICA-WC; (4) SICA-WHR), type I errors were generally set at 0·05, with main effect *P* values between 0·05 and 0·10 labelled as marginally significant, whereas a *P* value below 0·10 was considered significant for interaction terms, as was done in other studies (for example, Beydoun *et al.*^(^^26^^)^) before correction for multiple testing. Multiple testing correction was done using a familywise Bonferroni procedure, whereby a family was defined by adiposity outcome type (i.e. status (ECA) *v.* change (SICA))^(^^32^^)^. Within each outcome, two alternate measures were used, namely WC and WHR. The corrected statistical significance criterion for main effect *P* values was reduced to *P* = 0·05/2 = 0·025 (marginal significance: *P* = 0·10/2 = 0·050). Because of their lower statistical power compared with main effects, interaction terms' critical *P* values were reduced to 0·05^(^^33^^)^. All analyses (except for LCA) were performed using Stata version 11.0 (StataCorp LP)^(^^34^^)^.

## Results

### Study sample characteristics and gene SNP distribution

Study sample characteristics are summarised in Table 1. All examined SNP were in Hardy–Weinberg equilibrium (*P* > 0·05). Within the VDR gene, three SNP (*Bsm*I, *Apa*I, *Taq*I) were in LD (*r*^2^ > 0·5) while *Cdx*-2 SNP was independent. In fact, *Bsm*I and *Apa*I both occur in the intron separating exons 8 and 9^(^^35^^)^. In the megalin gene, rs4668123 and rs2075252 were in moderate LD (*r*^2^ 0·42) while rs3755166 was independent (*r*^2^ < 0·20) (online Supplementary material S1, Fig. S1(a) and Fig. S1(b)). For each SNP, one genotype had a relative frequency >40 % and thus was dominant compared with the other genotypes. SNPLC and SNPHAP distributions are also presented in online Supplementary material S1, Fig. S1(a) and Fig. S1(b).

**Table 1.** Study sample characteristics (Baltimore Longitudinal Study of Aging) (Number of subjects and percentages, mean values and standard deviations)

	*n*	%
Female	702	47·85
Age at first visit (years)	702	
Mean		52·34
sd		16·7
≤20	1	0·14
21–29	62	8·83
30–39	130	18·52
40–49	160	22·79
50–59	113	16·10
60–69	94	13·39
70–79	92	13·11
80 +	50	7·12
Education at first visit (years)	674	–
Mean		16·85
sd		2·53
Smoking status at first visit	644	
Never	254	39·44
Former	271	42·08
Current	119	18·48
Type 2 diabetes at first visit	702	1·28
Hypertension at first visit	689	27·00
CVD at first visit*	702	3·85
Dyslipidaemia at first visit	702	6·70
Central adiposity outcomes†		
ECA		
WC >100·40 cm (men); >88·80 cm (women)	631	22·7
WHR >0·968 (men); >0·846 (women)	631	22·5
SICA		
WC >0·448 cm (men); >0·583 (women)	631	30·9
WHR >0·0025 (men); >0·0038 (women)	631	34·2

ECA, elevated central adiposity; WC, waist circumference; WHR, waist:hip ratio; SICA, significant increase in central adiposity.

* Reported any of the following conditions at first visit: stroke, congestive heart failure, non-fatal myocardial infarction, or atrial fibrillation.

† WC and WHR were predicted at mean age at follow-up using a multivariate linear mixed model controlling for sex, race/ethnicity, education (years), and smoking status, with age added among the fixed-effect variables to allow for quadratic non-linear change. The slope or annual rate of change was predicted from these models at the mean age at follow-up (i.e. between age 50 years and individual mean age of follow-up for each central adiposity measure) (see online Supplementary Material S2 for more details).

### Vitamin D receptor and megalin SNP's associations with central adiposity

Table 2 examines, among others, the association between VDR SNP (entered alternatively, models 1·1–2·8) and central adiposity (ECA-WC, ECA-WHR, SICA-WC and SICA-WHR), using multiple logistic regression models. Most associations were non-significant, after Bonferroni correction (refer to type I error correction in Statistical analysis section). However, having a CC (*v*. AA) genotype on the *Apa*I:A/C SNP increased the risk of SICA-WHR (OR 1·76; 95 % CI 1·06, 2·92; *P* = 0·029) with a clear dose–response relationship with each A nucleotide (*P* for trend = 0·024).

**Table 2.** Vitamin D receptor (VDR) and Megalin gene SNP associations with predicted central adiposity outcomes: multiple logistic regression analysis (Baltimore Longitudinal Study of Aging) (Odds ratios and 95 % confidence intervals)

	Predicted central adiposity outcomes‡							
	ECA				SICA			
	*n*	OR§	95 % CI	*P* for trend	*n*	OR§	95 % CI	*P* for trend
VDR SNP: WC: models 1·1–1·8								
*VDR*: rs11568820 (CdX-2:T/C)	617				617			
TT = 0		1		0·415		1		0·456
CT = 1		0·88	0·29, 2·61			0·81	0·26, 2·49	
CC = 2		1·10	0·38, 3·14			1·01	0·34, 2·99	
*VDR*: rs1544410 (BsmI:G/A)	616				616			
GG = 0		1		0·346		1		0·694
GA = 1		1·05	0·68, 1·63			0·85	0·55, 1·31	
AA = 2		1·34	0·77, 2·35			0·94	0·53, 1·66	
*VDR*: rs7975232 (ApAI:A/C)	617				617			
AA = 0		1		0·670		1		0·064†
AC = 1		0·83	0·53, 1·31			1·26	0·80, 2·00	
CC = 2		0·91	0·53, 1·57			1·69†	0·97, 2·94	
*VDR*: rs731236 (TaqI:G/A)	617				617			
GG = 0		1		0·454		1		0·484
GA = 1		0·80	0·47, 1·35			0·92	0·53, 1·60	
AA = 2		0·78	0·45, 1·37			1·15	0·65, 2·04	
VDR SNP: WHR: models 2·1–2·8								
*VDR*: rs11568820 (CdX-2:T/C)	617				617			
TT = 0		1		0·363		1		0·891
CT = 1		0·80	0·27, 2·39			0·50	0·19, 1·32	
CC = 2		1·06	0·37, 3·01			0·62	0·24, 1·56	
*VDR*: rs1544410 (BsmI:G/A)	616				616			
GG = 0		1		0·976		1		0·071†
GA = 1		0·92	0·59, 1·44			0·79	0·53, 1·16	
AA = 2		1·04	0·58, 1·87			0·63†	0·37, 1·06	
*VDR*: rs7975232 (ApAI:A/C)	617				617			
AA = 0		1		0·821		1		0·024*
AC = 1		0·74	0·46, 1·17			1·53†	1·01, 2·34	
CC = 2		0·98	0·57, 1·70			1·76*	1·06, 2·92	
*VDR*: rs731236 (TaqI:G/A)	617				617			
GG = 0		1		0·906		1		0·038*
GA = 1		0·90	0·51, 1·57			1·26	0·76, 2·09	
AA = 2		1·00	0·56, 1·78			1·69†	1·00, 2·87	
Megalin SNP: WC: models 3·1–3·2	617				617			
*Megalin*: rs3755166:G/A				0·387				0·665
GG = 0		1				1		
GA = 1		1·06	0·64, 1·77			0·96	0·56, 1·63	
AA = 2		0·83	0·51, 1·32			1·09	0·69, 1·75	
*Megalin*: rs2075252:C/T	617			0·577	617			0·920
CC = 0		1				1		
CT = 1		0·69	0·43, 1·11			0·66†	0·42, 1·06	
TT = 2		1·08	0·57, 2·03			1·36	0·72, 2·56	
*Megalin*: rs4668123:C/T	609			0·730	609			
CC = 0		1				1		0·545
CT = 1		0·81	0·52, 1·25			0·91	0·60, 1·39	
TT = 2		1·74	0·82, 3·71			1·71	0·77, 3·77	
Megalin SNP: WHR: models 4·1–4·2								
*Megalin*: rs3755166:G/A	617				617			
GG = 0		1		0·184		1		0·585
GA = 1		0·88	0·52, 1·47			1·00	0·62, 1·61	
AA = 2		0·72	0·45, 1·17			0·89	0·58, 1·37	
*Megalin*: rs2075252:C/T	617				617			
CC = 0		1		0·042*		1		
CT = 1		1·08	0·68, 1·72			0·86	0·57, 1·30	0·367
TT = 2		2·14*	1·15, 3·99			1·59	0·89, 2·81	
*Megalin*: rs4668123:C/T	609				609			
CC = 0		1		0·093†		1		0·393
CT = 1		1·35	0·88, 2·08			0·95	0·65, 1·39	
TT = 2		1·69	0·75, 3·82			1·74	0·85, 3·55	

ECA, elevated central adiposity; SICA, significant increase in central adiposity; WC, waist circumference; WHR, waist:hip ratio.

* *P* < 0·05 for null hypothesis that Log_e_(OR) = 0.

† *P* < 0·10 for null hypothesis that Log_e_(OR) = 0.

‡ WC and WHR (ECA and SICA) were predicted using a linear mixed model controlling for sex, race/ethnicity, education (years), and smoking status, with age added among the fixed-effect variables to allow for quadratic non-linear change (see online Supplementary Material S2 for more details).

§ Based on multiple logistic regression models with outcome being ECA or SICA for WC or WHR and main exposures being each VDR SNP (models 1·1–1·8 or 2·1–2·8) or the three megalin SNP (models 3·1–4·2). The model controlled for first-visit age, mean age at follow-up, sex, education, first-visit smoking status, first-visit self-reported type 2 diabetes, hypertension, CVD and the two principal component analysis factor scores.

Similarly, when megalin SNP were entered simultaneously into models with each of the four outcomes (models 3·1–4·2), after correction for multiple testing, we found that the TT genotype contrasted with CC for Megalin:rs2075252:C/T was associated with a significantly higher odds of ECA-WHR (OR 2·14; 95 % CI 1·15, 3·99; *P* = 0·017) with a marginally significant dose–response relationship (*P* for trend = 0·042).

### Vitamin D receptor and Megalin SNP latent classes' associations with central adiposity

**Fig. 1.**
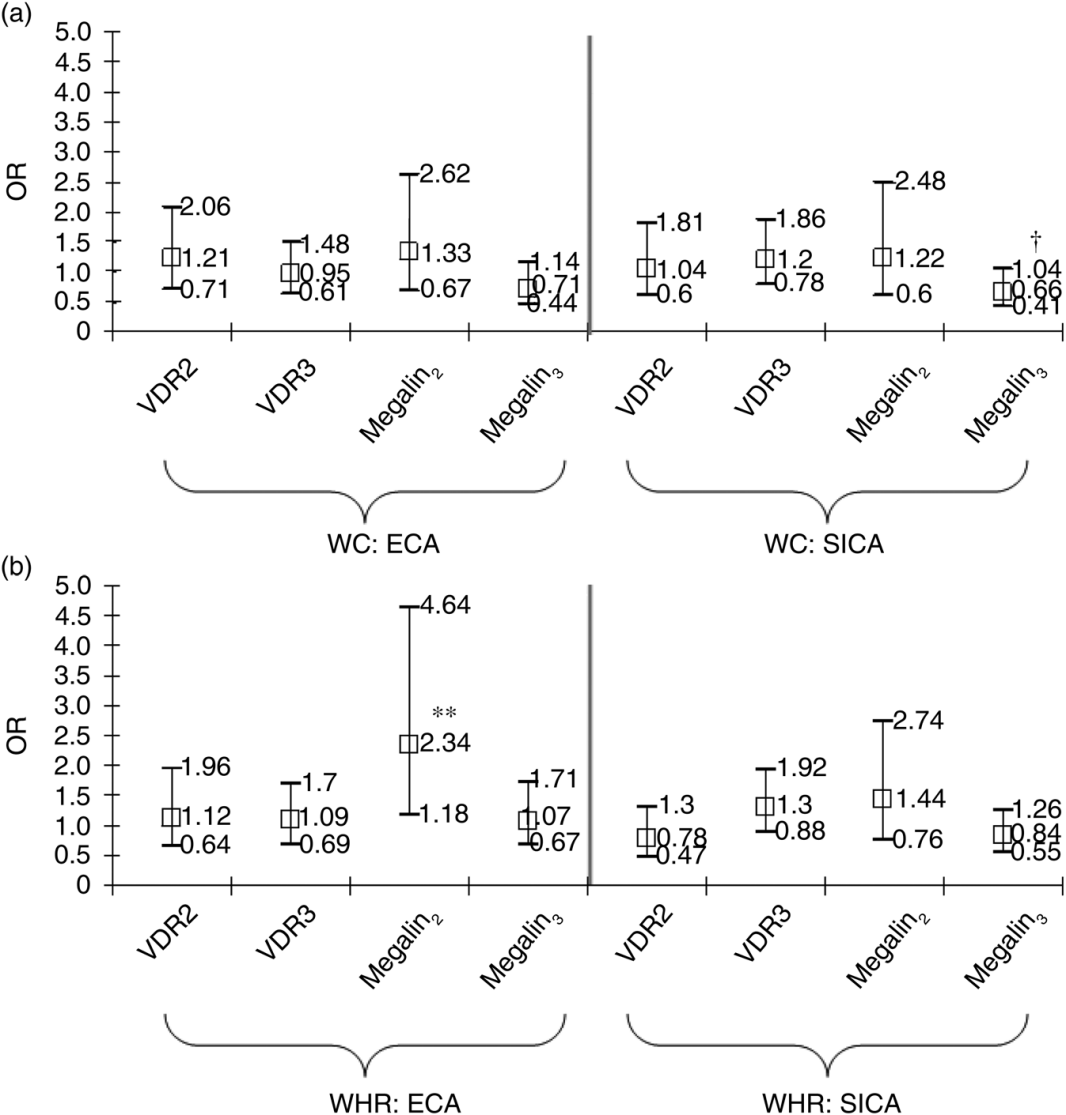
Associations of vitamin D receptor (VDR) and megalin SNP latent classes with elevated central adiposity (ECA) and significant increase in central adiposity (SICA), for waist circumference (WC) (a) and waist:hip ratio (WHR) (b): multiple logistic regression model. VDR and megalin SNP latent classes were entered together into the model as dummy variables. VDR_1_ and megalin_1_ SNP latent classes were taken as referent categories to which the other two SNP latent classes per gene were contrasted. The model was adjusted for covariates listed in Tables 2 and 3. See Methods section for description of the four outcomes and the SNP latent classes. Values are odds ratios, with 95 % CI represented by vertical bars. ** *P* < 0·025 for null hypothesis that Log_e_(OR) = 0. † *P* < 0·10 for null hypothesis that Log_e_(OR) = 0.

Using LCA, three SNPLC per gene were created. One key finding emerged for SNPLC related to central adiposity in the total population (Fig. 1(a) and Fig. 1(b)). Comparing each minor SNPLC with the most dominant one, we found that Megalin_2_ *v.* Megalin_1_ was associated with significantly increased odds of ECA-WHR in the total population (OR 2·34; 95 % CI 1·18, 4·64; *P* = 0·015), which remained significant after correction for multiple testing (Fig. 1(b)).

**Table 3.** Vitamin D receptor (VDR) and Megalin gene SNP latent class (SNPLC) associations with predicted central adiposity outcomes, stratified by sex: multiple logistic regression analysis (Baltimore Longitudinal Study of Aging)‡ (Odds ratios and 95 % confidence intervals)

	Men				Women			
	*n*	OR§	95 % CI	*P*	*n*	OR§	95 % CI	*P*
ECA								
WC	368				245			
*VDR*_*2*_ *v. VDR*_*1*_		1·32	0·67, 2·60	0·428		0·92	0·37, 2·27	0·854
*VDR*_*3*_ *v. VDR*_*1*_		1·24	0·70, 2·17	0·460		0·56	0·26, 1·19	0·132
*Megalin*_*2*_ *v. Megalin*_*1*_		1·59	0·65, 3·89	0·307		1·17	0·38, 3·59	0·788
*Megalin*_*3*_ *v. Megalin*_*1*_		0·60	0·32, 1·07	0·101		0·94	0·43, 2·07	0·882
WHR	367				253			
*VDR*_*2*_ *v. VDR*_*1*_		0·84	0·40, 1·76	0·653		1·91	0·72, 5·10	0·197
*VDR*_*3*_ *v. VDR*_*1*_		0·85	0·48, 1·51	0·585		1·73	0·77, 3·89	0·180
*Megalin*_*2*_ *v. Megalin*_*1*_		2·87	1·15, 7·12	0·023**		2·44	0·76, 7·82	0·133
*Megalin*_*3*_ *v. Megalin*_*1*_		0·96	0·53, 1·75	0·906		1·48	0·65, 3·40	0·350
SICA								
WC	368				245			
*VDR*_*2*_ *v. VDR*_*1*_		1·36	0·67, 2·77	0·395		0·68	0·26, 1·79	0·438
*VDR*_*3*_ *v. VDR*_*1*_		1·28	0·72, 2·27	0·398		1·12	0·55, 2·28	0·747
*Megalin*_*2*_ *v. Megalin*_*1*_		1·22	0·45, 3·32	0·699		1·17	0·40, 3·46	0·771
*Megalin*_*3*_ *v. Megalin*_*1*_		0·48	0·26, 0·88	0·019**		1·18	0·55, 2·53	0·673
WHR	368				243			
*VDR*_*2*_ *v. VDR*_*1*_		0·53	0·26, 1·05	0·069†		1·18	0·49, 2·83	0·711
*VDR*_*3*_ *v. VDR*_*1*_		1·05	0·63, 1·76	0·842		2·08	1·03, 4·17	0·040*
*Megalin*_*2*_ *v. Megalin*_*1*_		1·55	0·64, 3·78	0·333		1·67	0·57, 4·90	0·351
*Megalin*_*3*_ *v. Megalin*_*1*_		0·67	0·39, 1·16	0·153		1·47	0·71, 3·03	0·300

ECA, elevated central adiposity; WC, waist circumference; WHR, waist:hip ratio; SICA, significant increase in central adiposity.

Significance for null hypothesis that Log_e_(OR) = 0: * *P* < 0·05, ** *P* < 0·025.

† *P* < 0·10 for null hypothesis that Log_e_(OR) = 0.

‡ WC and WHR (ECA and SICA) were predicted using a linear mixed model controlling for sex, race/ethnicity, education (years), and smoking status, with age added among the fixed-effect variables to allow for quadratic non-linear change (see online Supplementary Material S2 for more details).

§ Based on multiple logistic regression models with outcome being ECA or SICA for WC or WHR and main exposures being VDR and megalin SNPLC entered simultaneously into the model for each outcome, stratifying by sex. The model controlled for first-visit age, mean age at follow-up, education, first-visit smoking status, first-visit self-reported type 2 diabetes, hypertension, CVD and the two principal component analysis factor scores.

### Vitamin D receptor and Megalin SNP latent classes' associations with central adiposity: sex-stratified findings

In Table 3, we conducted similar regression models as in Fig. 1(a) and Fig. 1(b), but stratifying by sex. Although sex differences were not statistically significant when testing sex × SNPLC interaction terms in separate models (*P* > 0·05), some of the significant associations that were detected in the total population were restricted only to men (Megalin_2_ *v.* Megalin_1_ (ECA-WHR): OR 2·87; 95 % CI 1·15, 7·12; *P* = 0·023; and Megalin_3_ *v.* Megalin_1_ (SICA-WC): OR 0·48; 95 % CI 0·26, 0·88; *P* = 0·019) and retained significance after correction for multiple testing. However, none of the VDR SNPLC associations with central adiposity were significant after this type of correction (*P* > 0·025).

### Vitamin D receptor *and Megalin SNP halotypes' associations with central adiposity*

**Fig. 2.**
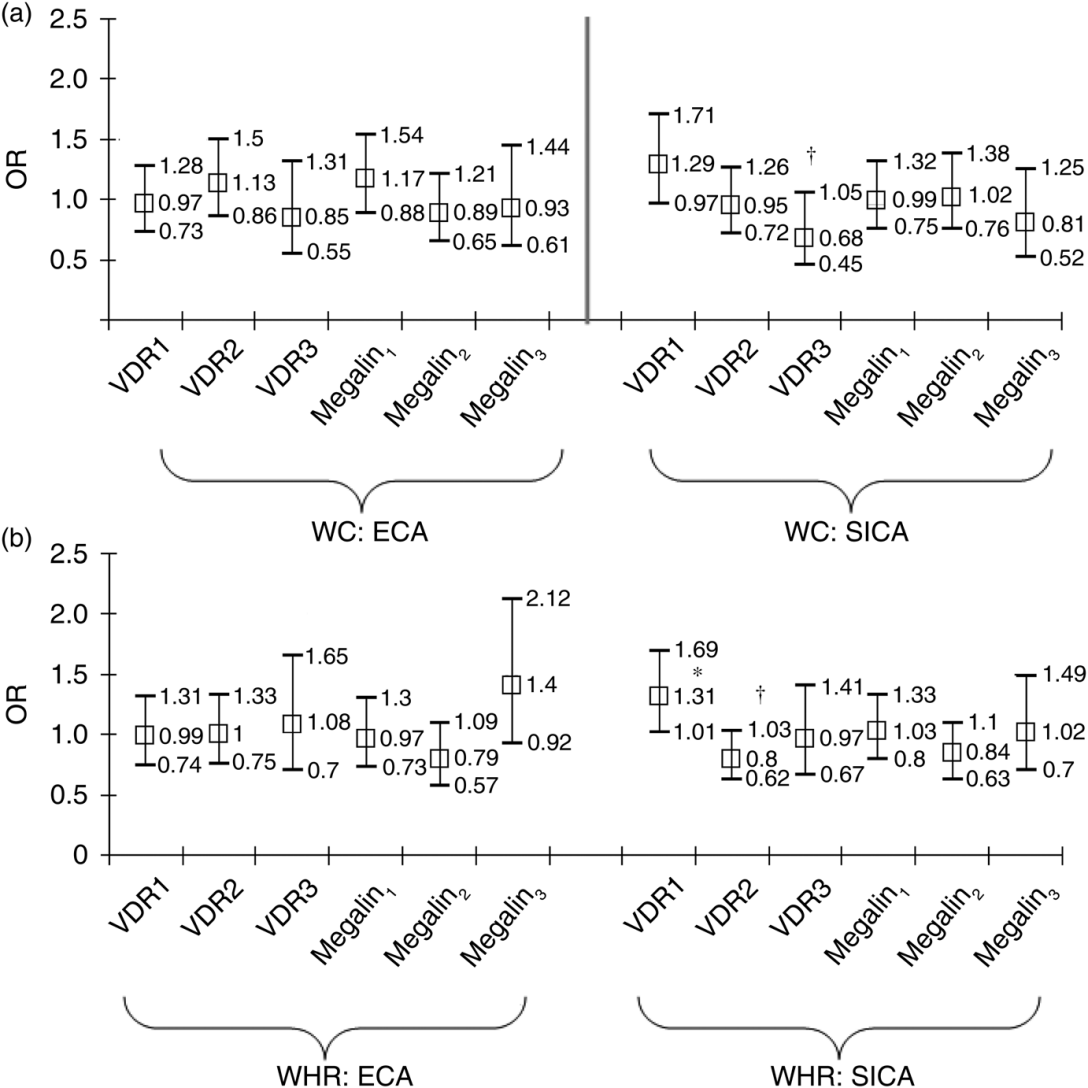
Associations of vitamin D receptor (VDR) and megalin SNP haplotypes with elevated central adiposity (ECA) and significant increase in central adiposity (SICA), for waist circumference (WC) (a) and waist:hip ratio (WHR) (b): multiple logistic regression model. VDR and megalin SNP haplotypes were entered separately into the model as an ordinal variable (0, 1, 2). The model was adjusted for covariates listed in Tables 2 and 3. See Methods section for description of the four outcomes and the SNP haplotypes. Values are odds ratios, with 95 % CI represented by vertical bars. * *P* < 0·05 for null hypothesis that Log_e_(OR) = 0. † *P* < 0·10 for null hypothesis that Log_e_(OR) = 0.

Using haplotype analysis, three SNPHAP per gene were created. Each of those six SNPHAP was entered separately in the main multiple logistic regression models as an ordinal variable. In both sexes combined, VDR_1_ SNPHAP was associated with an increased odds of SICA-WHR (OR 1·31; 95 % CI 1·01, 1·69; *P* = 0·038), though significance was not retained after correction for multiple testing (Fig. 2(a) and Fig. 2(b)).

### Vitamin D receptor and Megalin SNP halotypes' associations with central adiposity: sex-stratified findings

Our sex-stratified analysis with SNPHAP (Table 4) uncovered many important findings, particularly for VDR SNPHAP. Among those key findings, VDR_3_ SNPHAP was associated with a lower odds of ECA-WC in women only (OR 0·37; 95 % CI 0·16, 0·87; *P* = 0·023) with significant sex differences (*P* < 0·05 for sex × SNPHAP interaction term). This finding was replicated for SICA-WC in women (OR 0·40; 95 % CI 0·19, 0·87; *P* = 0·020), though without significant sex differences. Moreover, among women only, VDR_1_ was related to an increased odds of SICA-WC (OR 1·87; 95 % CI 1·14, 3·07; *P* = 0·014) without significant sex differentials. Those associations retained statistical significance upon correction for multiple testing, which was not the case of Megalin SNPHAP (*P* > 0·025).

**Table 4.** Vitamin D receptor (VDR) and Megalin gene SNP haplotype (SNPHAP) associations with predicted central adiposity outcomes, stratified by sex: multiple logistic regression analysis (Baltimore Longitudinal Study of Aging)§ (Odds ratios and 95 % confidence intervals)

	Men				Women			
	*n*	OR‖	95 % CI	*P*	*n*	OR‖	95 % CI	*P*
ECA								
WC	364				236			
*VDR*_*1*_, *GCA* (0, 1, 2)		0·89	0·62, 1·28	0·534		1·15	0·71, 1·87	0·562
*VDR*_*2*_, *AAG* (0, 1, 2)		1·04	0·73, 1·50	0·817		1·30	0·82, 2·06	0·256
*VDR*_*3*_, *GAA* (0, 1, 2)		1·25	0·73, 2·13	0·417		0·37	0·16, 0·87	0·023**‡
*Megalin*_*1*_, *GCC* (0, 1, 2)		1·00	0·69, 1·44	0·995		1·41	0·89, 2·23	0·145
*Megalin*_*2*_, *ACC* (0, 1, 2)		1·13	0·77, 1·66	0·538		0·62	0·36, 1·05	0·076†
*Megalin*_*3*_, *GTT* (0, 1, 2)		0·95	0·54, 1·68	0·862		0·97	0·49, 1·93	0·927
WHR	363				236			
*VDR*_*1*_, *GCA* (0, 1, 2)		1·01	0·70, 1·46	0·959		0·96	0·56, 1·61	0·894
*VDR*_*2*_, *AAG* (0, 1, 2)		1·06	0·73, 1·53	0·759		0·90	0·55, 1·48	0·692
*VDR*_*3*_, *GAA* (0, 1, 2)		0·92	0·52, 1·63	0·764		1·38	0·66, 2·88	0·393
*Megalin*_*1*_, *GCC* (0, 1, 2)		1·07	0·74, 1·55	0·730		0·84	0·51, 1·37	0·485
*Megalin*_*2*_, *ACC* (0, 1, 2)		0·92	0·61, 1·37	0·674		0·55	0·30, 1·00	0·049*
*Megalin*_*3*_, *GTT* (0, 1, 2)		1·66	0·95, 2·88	0·075†		1·41	0·70, 2·82	0·332
SICA								
WC	364				242			
*VDR*_*1*_, *GCA* (0, 1, 2)		1·08	0·74, 1·56	0·692		1·87	1·14, 3·07	0·014**
*VDR*_*2*_, *AAG* (0, 1, 2)		1·02	0·71, 1·49	0·895		0·88	0·56, 1·37	0·568
*VDR*_*3*_, *GAA* (0, 1, 2)		0·84	0·49, 1·45	0·535		0·40	0·19, 0·87	0·020**
*Megalin*_*1*_, *GCC* (0, 1, 2)		0·89	0·61, 1·30	0·552		1·08	0·68, 1·71	0·746
*Megalin*_*2*_, *ACC* (0, 1, 2)		1·29	0·87, 1·91	0·212		0·76	0·46, 1·25	0·278
*Megalin*_*3*_, *GTT* (0, 1, 2)		0·72	0·40, 1·31	0·286		1·00	0·52, 1·90	0·990
WHR	364				234			
*VDR*_*1*_, *GCA* (0, 1, 2)		1·34	0·96, 1·88	0·090†		1·52	0·97, 2·38	0·065†
*VDR*_*2*_, *AAG* (0, 1, 2)		0·77	0·55, 1·09	0·138		0·74	0·48, 1·13	0·165
*VDR*_*3*_, *GAA* (0, 1, 2)		0·97	0·59, 1·60	0·903		0·89	0·47, 1·72	0·739
*Megalin*_*1*_, *GCC* (0, 1, 2)		1·05	0·75, 1·48	0·760		0·85	0·55, 1·32	0·475
*Megalin*_*2*_, *ACC* (0, 1, 2)		0·86	0·60, 1·24	0·414		0·82	0·51, 1·31	0·406
*Megalin*_*3*_, *GTT* (0, 1, 2)		0·83	0·49, 1·43	0·510		1·43	0·79, 2·60	0·237

ECA, elevated central adiposity; WC, waist circumference; WHR, waist:hip ratio; SICA, significant increase in central adiposity.

Significance for null hypothesis that Log_e_(OR) = 0: *P < 0·05, **P < 0·025.

† *P* < 0·10 for null hypothesis that Log_e_(OR) = 0.

‡*P* < 0·05 for null hypothesis that sex × SNPHAP interaction term = 0 in a model where main effect of sex was added.

§ WC and WHR were predicted at mean age at follow-up using a multivariate linear mixed model controlling for sex, race/ethnicity, education (years), and smoking status, with age added among the fixed-effect variables to allow for quadratic non-linear change. The slope or annual rate of change was predicted from these models at the mean age at follow-up (i.e. between age 50 years and individual mean age of follow-up for each central adiposity measure) (see online Supplementary Material S2 for more details).

║ Based on multiple logistic regression models with outcome being ECA or SICA for WC or WHR and main exposures being VDR and megalin SNPHAP entered simultaneously into the model for each outcome, stratifying by sex. The model controlled for first-visit age, mean age at follow-up, education, first-visit smoking status, first-visit self-reported type 2 diabetes, hypertension, CVD and the two principal component analysis factor scores.

## Discussion

The present study examined longitudinal associations of VDR and megalin gene polymorphisms with central adiposity, using extensive data from the BLSA, an ongoing prospective open cohort study. Study participants consisted of non-Hispanic white adults residing in Baltimore city, with one or more visits at age ≥50 years, and complete data (*n* 609–617). Available repeated assessments on WC and WHR were used to form four binary outcomes, which were defined by multiple linear mixed models, mid-follow-up age estimators for central adiposity level and annual rate of change with cut-points set at the sex-specific 80th percentile: ECA-WC and ECA-WHR, and SICA-WC and SICA-WHR.

Selected SNP for VDR (four SNP: (1) rs11568820 (CdX-2:T/C); (2) rs1544410 (BsmI:G/A); (3) rs7975232 (ApaI:A/C); (4) rs731236 (TaqI:G/A)) and Megalin (three SNP: (1) rs3755166:G/A; (2) rs2075252:C/T; (3) rs4668123:C/T) were included as main exposures, from which SNPLC and SNPHAP were created. Multiple logistic regression analyses indicated that, in men, higher ECA-WHR odds were associated with SNPLC Megalin_2_:rs3755166[–]/rs2075252[TT]/rs4668123[T–] (*v*. Megalin_1_:rs3755166[–]/rs2075252[CC]/rs4668123[–]) (OR 2·87; 95 % CI 1·15, 7·12; *P* = 0·023) and that SNPLC Megalin_3_:rs3755166[–]/rs2075252[CT]/rs4668123[–] (*v*. Megalin_1_) was linked to lower SICA-WC odds (OR 0·48; 95 % CI 0·26, 0·88; *P* = 0·019) (*P* > 0·05 for sex × SNPLC). In women, VDR_3_ SNPHAP (GAA:bAT) was related to lower odds of ECA-WC (OR 0·37; 95 % CI 0·16, 0·87; *P* = 0·023) (*P* < 0·05 for sex × SNPHAP), VDR_1_ SNPHAP (GCA:baT) was associated with greater odds and VDR_3_ SNPHAP (GAA:bAT) with lower odds of SICA-WC (*P* > 0·05 for sex × SNPHAP).

Several recent cross-sectional and case–control studies have examined VDR genetic polymorphisms as potential risk markers for central adiposity and related metabolic disorders^(^^9^^–^^11^^,^^16^^,^^17^^,^^36^^,^^37^^)^. When testing VDR SNP associations with adiposity, a recent cross-sectional study (176 randomly selected men aged 25–65 years) found that homozygous BsmI (AA *v.* GG) was associated with higher BMI (29·0 *v.* 26·8 kg/m^2^; *P* = 0·024) and higher WC (101·8 *v.* 96·2 cm; *P* = 0·014)^(^^11^^)^. A similar finding was observed in another cross-sectional study of 175 women with body weight and fat mass as two outcomes and the VDR SNP *Bsm*I being of interest^(^^10^^)^. In a more recent study with a larger sample size (*n* 1773) of women, the association of fourteen VDR SNP with three adiposity measures was examined, including WC. Results suggested that the homozygous rare variant of rs3782905 found in the 3′ VDR region (LD between rs3782905 and *Bsm*I in Caucasian HapMap is about 0·42) was associated on average with 4·4 cm larger WC compared with the homozygous common variant (Bonferroni-adjusted *P* = 0·004)^(^^9^^)^. These consistent findings for *Bsm*I and obesity with the risk increasing allele being ‘A’ were confirmed when the phenotypes of interest were type 2 diabetes, fasting glucose level and CHD risk in recent studies^(^^13^^,^^14^^)^. In contrast, among 351 postmenopausal healthy women, VDR *Bsm*I polymorphism (‘A’ risk allele) was not associated with obesity or insulin resistance but was connected with an unfavourable lipid profile^(^^36^^)^. Additionally, a case–control study with 309 unrelated French subjects with type 2 diabetes, and among those with early onset in particular, the *Taq*I SNP (‘A’ allele) was associated with a higher BMI and an increased prevalence of obesity, compared with the controls^(^^16^^)^. The *Cdx-*2 SNP was related only to BMI, fat mass and percentage fat mass in one study of 1215 subjects from 400 Chinese nuclear families^(^^17^^)^. In the present study, only the *Apa*I SNP (‘C’ allele dosage) appeared to significantly increase the odds of SICA-WHR (*P* for trend = 0·024).

Few previous studies have examined VDR SNPHAP (in addition to SNP) as predictors of adiposity, and none found significant associations (for example, Gu *et al.*^(^^17^^)^). However, other studies examined VDR SNPHAP in relation to diabetes (types 1 and 2), insulin resistance and cardiovascular outcomes. For instance, in a population-based study of men and women aged 55–80 years, each copy of the baT haplotype was associated with a 20 % increased likelihood of electrocardiogram-confirmed myocardial infarction, after adjustment for established CVD risk factors^(^^38^^)^. The latter finding suggests that baT, which we found to increase the risk of longitudinal increase in WC among women, may also be a risk factor for cardiovascular events.

Although no prior research had tested the association between VDR SNPLC and megalin (SNP, SNPHAP or SNPLC) and adiposity, we found that they might be important risk factors for central adiposity (ECA and SICA), in some cases only in one sex. Thus, our findings need to be replicated in larger samples of adult men and women with repeated measures on WC and WHR before efforts to uncover potential reasons for sex differences can be made.

Although the exact mechanism is unknown, evidence supports the role of vitamin D on adiposity. *In vitro* studies have shown that vitamin D stabilises VDR and suppresses adipocyte differentiation through C/EBPα (cytidine-cytidine-adenosine-adenosine-thymidine (CCAAT)/enhancer binding protein α) inhibition and PPARγ expression and activity^(^^39^^)^. VDR overexpression inhibited adipocyte differentiation independently of vitamin D, suggesting that the VDR plays a crucial role in adipocyte maturation. If the polymorphisms in VDR and megalin are functional or are tagging SNP that alter the availability or activity of vitamin D, it is conceivable that these SNP influence adiposity traits through the regulation of adipocyte differentiation. The *Cdx*-2 polymorphism in VDR has been shown to have an effect on VDR activity while the *Taq*I-*Apa*I-*Bsm*I SNP in the 3′ untranslated region of the gene is thought to be a tag SNP^(^^40^^)^. In the present study, the 3′ region SNP were more strongly associated with longitudinal adiposity trajectory; therefore fine mapping of this region to identify the functional SNP may provide insight into the mechanism by which VDR SNP regulate adiposity traits. The two non-synonymous SNP in megalin (rs2075252, rs3668123) were driving the association between megalin and adiposity traits^(^^41^^)^. Whether these coding SNP influence megalin gene function is unknown. However, if functional, these SNP could alter vitamin D availability in cells and thereby regulate adipocyte differentiation.

The present study has several strengths. It included a large number of consecutive visits per participant, and made use of advanced statistical techniques by combining linear mixed models with multiple logistic regression analyses^(^^42^^)^ to examine associations between gene SNP, SNPLC (defined using LCA) and SNPHAP (defined using haplotype analysis) and four measures of central adiposity status and change.

However, in light of some limitations, findings of the present study must be interpreted with caution. Indeed, the BLSA is an open cohort study of a convenience sample of participants, experiencing continuous recruitment and dropout. Moreover, genetic data were available only for a subset of the initial cohort, yielding a smaller sample size and a younger mean age. To reduce selection biases resulting from this sampling scheme, we used a number of statistical techniques, including a two-stage Heckman selection model. Further, even though observation frequency for central adiposity was high (mean about five visits), the data structure was largely unbalanced given that first-visit age and duration between visits varied across participants. Consequently, we used mixed models to predict the continuous version of the four adiposity outcomes at mean follow-up age. Our main statistical models also controlled for mean follow-up and first-visit age. Moreover, no data were readily available on potential confounders including serum vitamin D, dietary intakes of Ca and vitamin D; physical activity particularly outdoors exercise which is a main determinant of vitamin D status and is also one of the main protective lifestyle factors related to obesity; alcohol or drug use and use of medications. Finally, we cannot rule out chance, residual confounding or selection bias for positive findings, particularly for the sex-specific analyses, and lack of power for negative findings. Thus, until those findings are further replicated in another independent sample, they should be interpreted with caution.

In conclusion, the key findings of the present study point to a relationship between VDR and megalin gene polymorphisms and central adiposity. To our knowledge this is the first study to examine longitudinal change in central adiposity in relation to polymorphisms in those two genes. However, further research is needed to replicate those findings in different populations, including populations of other racial and ethnic groups, in order to confirm the biological significance of those polymorphisms in relation to central adiposity phenotypes.

## Supplementary Material

Supplementary Material Supplementary information supplied by authors.

Supplementary Material Supplementary information supplied by authors.

Supplementary Material Supplementary information supplied by authors.

## Supplementary material

To view supplementary material for this article, please visit http://dx.doi.org/10.1017/jns.2013.19
