# Supplementary material for: Vitamin D receptor and megalin gene polymorphisms are associated with central adiposity status and changes among US adults
Source: J Nutr Sci. 2013 Oct 30;2:e33. doi: 10.1017/jns.2013.19 (PMC4153078; doi:10.1017/jns.2013.19)
Supplement: Supplementary Material — Supplementary information supplied by authors. [file S2048679013000190sup001.ppt]

## Slide 1
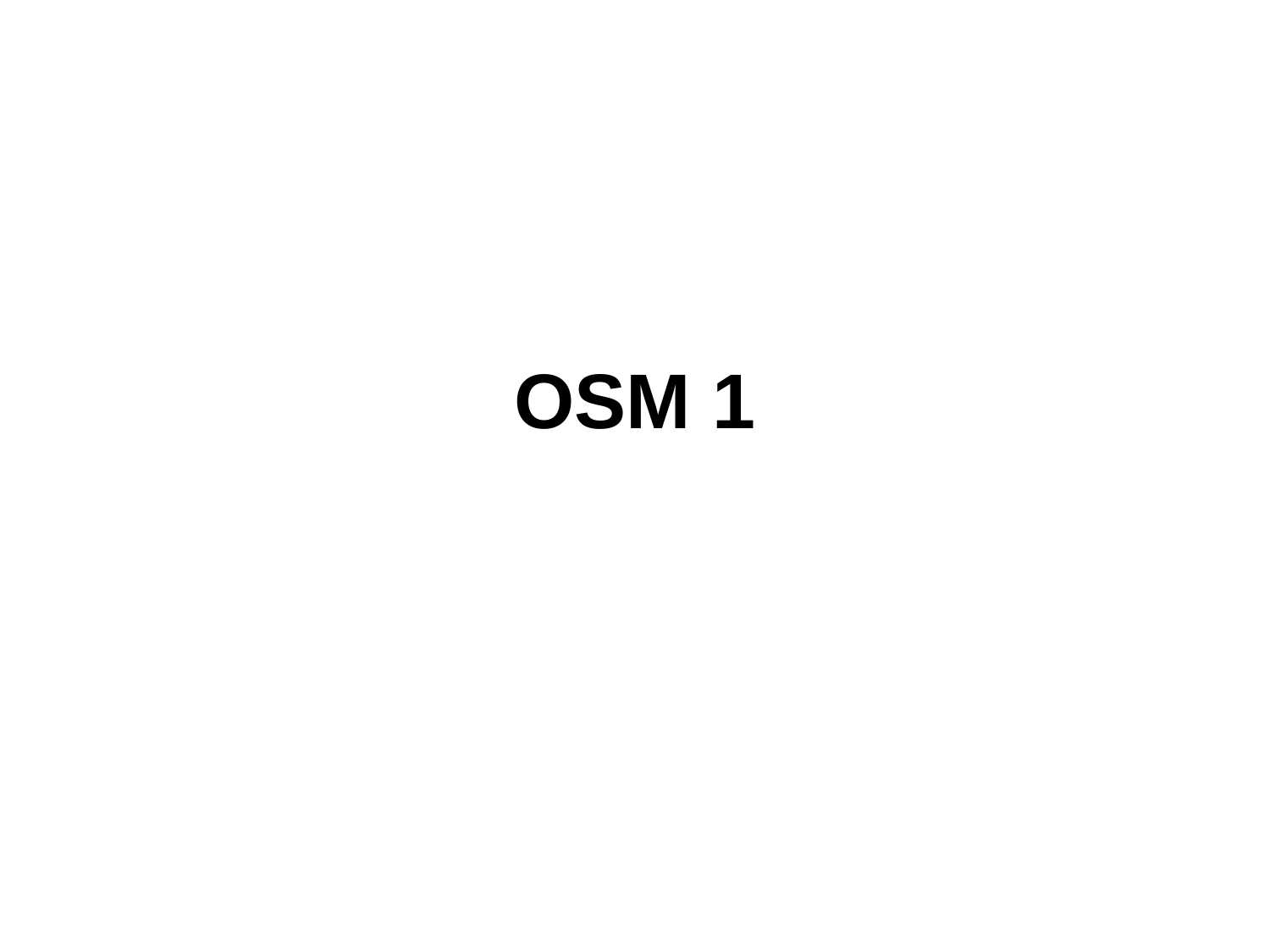

# OSM 1

## Slide 2
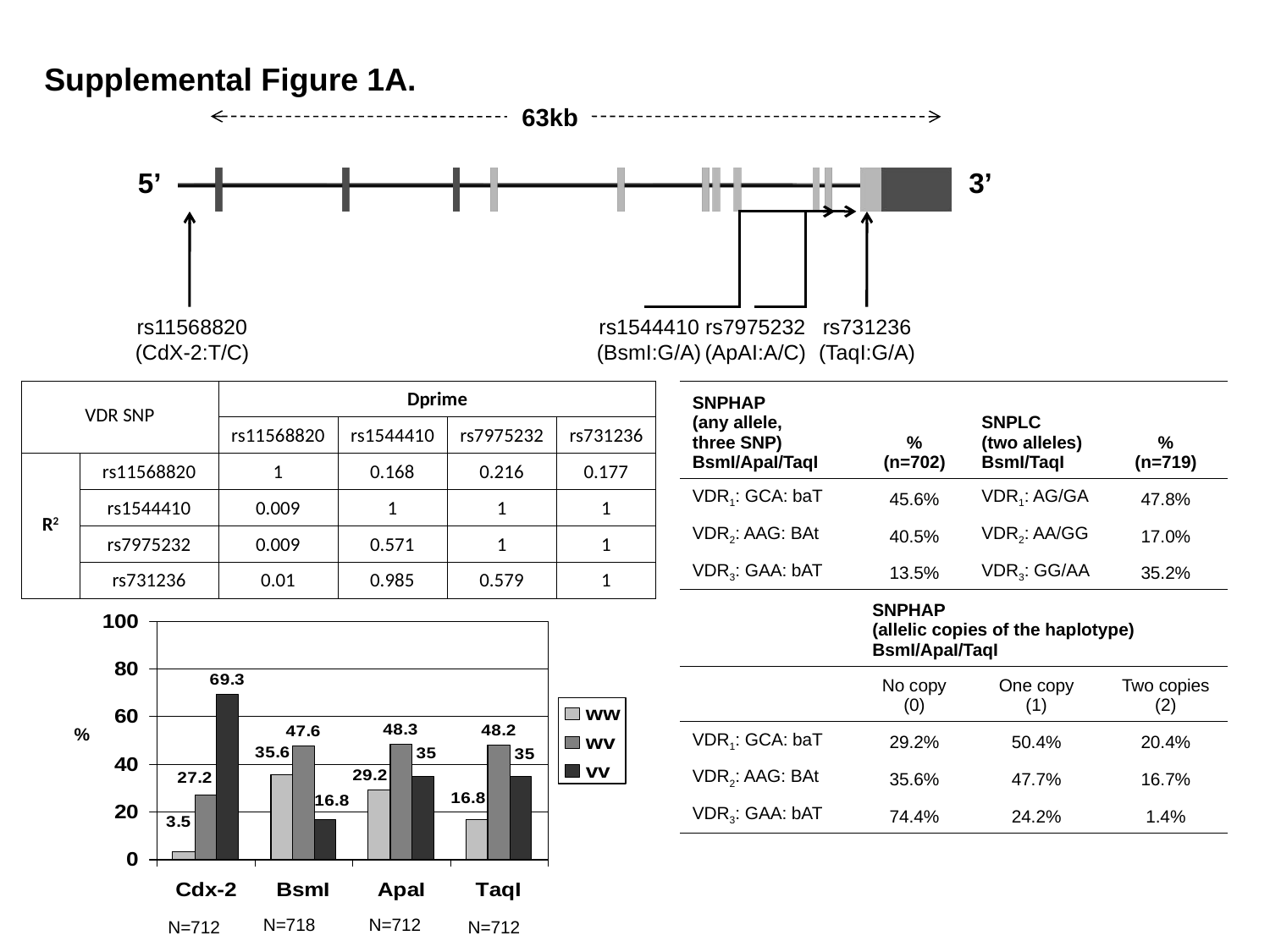

Supplemental Figure 1A.
63kb
5’
rs11568820
(CdX-2:T/C)
rs1544410
(BsmI:G/A)
rs7975232
(ApAI:A/C)
rs731236
(TaqI:G/A)
3’
| VDR SNP | | Dprime | | | |
| --- | --- | --- | --- | --- | --- |
| | | rs11568820 | rs1544410 | rs7975232 | rs731236 |
| R2 | rs11568820 | 1 | 0.168 | 0.216 | 0.177 |
| | rs1544410 | 0.009 | 1 | 1 | 1 |
| | rs7975232 | 0.009 | 0.571 | 1 | 1 |
| | rs731236 | 0.01 | 0.985 | 0.579 | 1 |
| SNPHAP (any allele, three SNP) BsmI/ApaI/TaqI | % (n=702) | SNPLC (two alleles) BsmI/TaqI | % (n=719) |
| --- | --- | --- | --- |
| VDR1: GCA: baT | 45.6% | VDR1: AG/GA | 47.8% |
| VDR2: AAG: BAt | 40.5% | VDR2: AA/GG | 17.0% |
| VDR3: GAA: bAT | 13.5% | VDR3: GG/AA | 35.2% |
| | SNPHAP (allelic copies of the haplotype) BsmI/ApaI/TaqI | | |
| | No copy (0) | One copy (1) | Two copies (2) |
| VDR1: GCA: baT | 29.2% | 50.4% | 20.4% |
| VDR2: AAG: BAt | 35.6% | 47.7% | 16.7% |
| VDR3: GAA: bAT | 74.4% | 24.2% | 1.4% |
| | | | |
| | | | |
| | | | |
N=718
N=712
N=712
N=712
%

## Slide 3
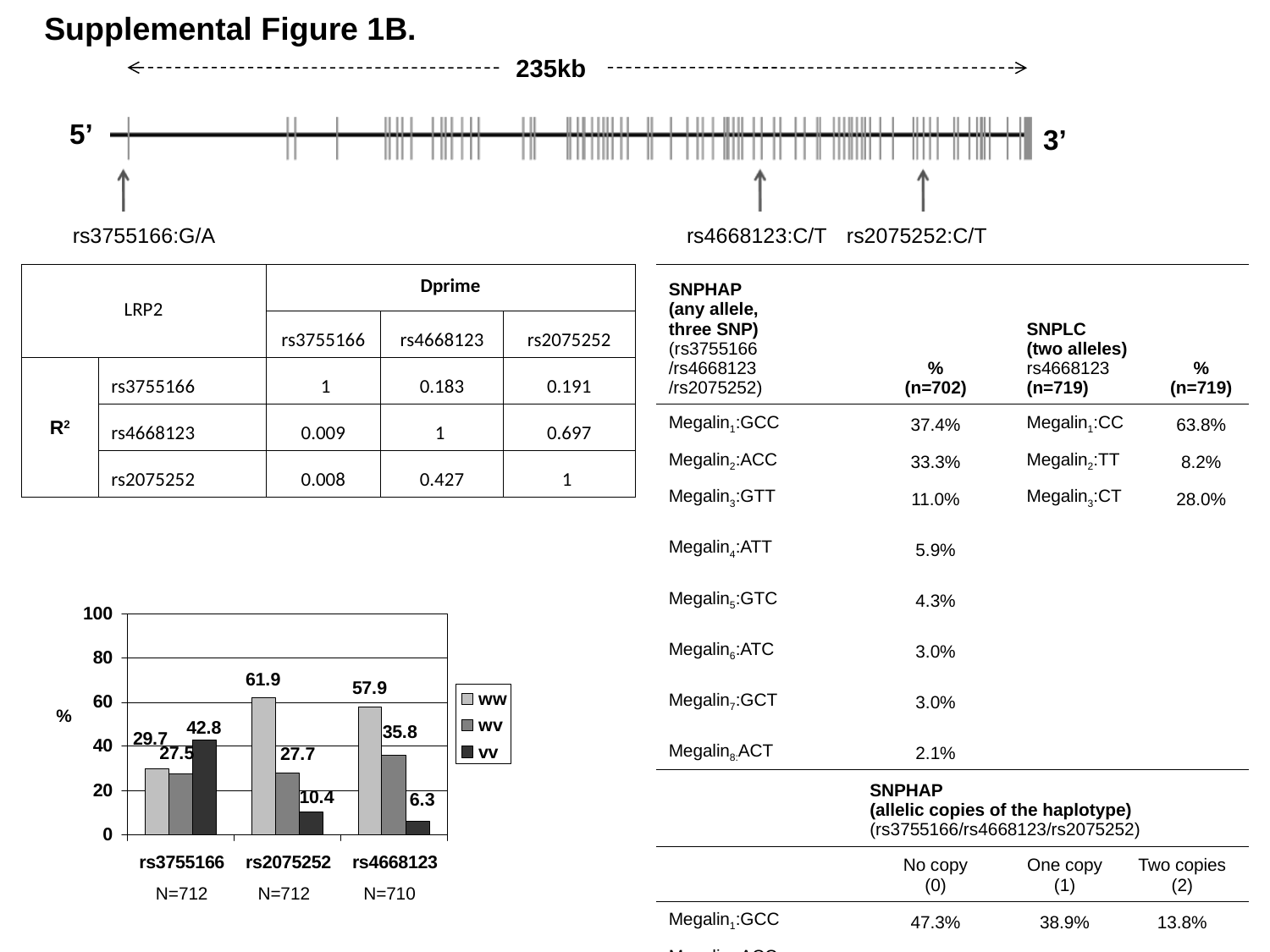

Supplemental Figure 1B.
235kb
5’
3’
rs3755166:G/A
rs4668123:C/T
rs2075252:C/T
| LRP2 | | Dprime | | |
| --- | --- | --- | --- | --- |
| | | rs3755166 | rs4668123 | rs2075252 |
| R2 | rs3755166 | 1 | 0.183 | 0.191 |
| | rs4668123 | 0.009 | 1 | 0.697 |
| | rs2075252 | 0.008 | 0.427 | 1 |
| SNPHAP (any allele, three SNP) (rs3755166 /rs4668123 /rs2075252) | % (n=702) | SNPLC (two alleles) rs4668123 (n=719) | | % (n=719) |
| --- | --- | --- | --- | --- |
| Megalin1:GCC | 37.4% | Megalin1:CC | | 63.8% |
| Megalin2:ACC | 33.3% | Megalin2:TT | | 8.2% |
| Megalin3:GTT | 11.0% | Megalin3:CT | | 28.0% |
| Megalin4:ATT | 5.9% | | | |
| Megalin5:GTC | 4.3% | | | |
| Megalin6:ATC | 3.0% | | | |
| Megalin7:GCT | 3.0% | | | |
| Megalin8:ACT | 2.1% | | | |
| | SNPHAP (allelic copies of the haplotype) (rs3755166/rs4668123/rs2075252) | | | |
| | No copy (0) | One copy (1) | Two copies (2) | |
| Megalin1:GCC | 47.3% | 38.9% | 13.8% | |
| Megalin2:ACC | 35.5% | 53.4% | 11.1% | |
| Megalin3:GTT | 74.1% | 24.2% | 1.7% | |
| | | | | |
| | | | | |
| | | | | |
N=712
N=712
N=710
%
