# Supplementary material for: Vitamin D receptor and megalin gene polymorphisms are associated with central adiposity status and changes among US adults
Source: J Nutr Sci. 2013 Oct 30;2:e33. doi: 10.1017/jns.2013.19 (PMC4153078; doi:10.1017/jns.2013.19)
Supplement: Supplementary Material — Supplementary information supplied by authors. [file S2048679013000190sup002.doc]

**OSM2**

**Linear mixed models for prediction of central adiposity**

A standard taxonomy of models (1) was used, starting from the unconditional means model (Model A), unconditional growth model (Model B), growth model with level-2 controlled effects of other factors namely sex, race/ethnicity, education and smoking status (Model C), and ending with a growth model with level-2 controlled effects of other factors, adding a squared-age term that would allow the rate of change to vary with time (Model D). In all models, age was centered at 50 years, while education was centered at 16 years. The following equations apply to each of the models considered:

| **Model** | **Level-1 model** | **Level-2 model** | **Composite model** |
| --- | --- | --- | --- |
| A | $Y_{ij}=\pi_{0i}+\varepsilon_{ij}$ | $\pi_{0i}=\gamma_{00}+\zeta_{0i}$ | $Y_{ij}=\gamma_{00}+(\zeta_{0i}+\varepsilon_{ij})$ |
| B | $Y_{ij}=\pi_{0i}+\pi_{1i}Age_{50}+\varepsilon_{ij}$ | $\pi_{0i}=\gamma_{00}+\zeta_{0i}$ $\pi_{1i}=\gamma_{10}+\zeta_{1i}$ | $Y_{ij}=\gamma_{00}+\gamma_{10}Age_{50}+(\zeta_{0i}+\zeta_{1i}Age_{50}+\varepsilon_{ij})$ |
| C | $Y_{ij}=\pi_{0i}+\pi_{1i}Age_{50}+\varepsilon_{ij}$ | $\pi_{0i}=\gamma_{00}+\sum_{k=1}^{7} \gamma_{0k}Z_{ik}+\zeta_{0i}$ $\pi_{1i}=\gamma_{10}+\sum_{k=1}^{7} \gamma_{1k}Z_{ik}+\zeta_{1i}$ | $\begin{matrix} Y_{ij}=\gamma_{00}+\sum_{k=1}^{7} \gamma_{0k}Z_{ik}+\gamma_{10}Age_{50}+\sum_{k=1}^{7} \gamma_{1k}Z_{ik}Age_{50} \\ +(\zeta_{0i}+\zeta_{1i}Age_{50}+\varepsilon_{ij}) \end{matrix}$ |
| D | $Y_{ij}=\pi_{0i}+\pi_{1i}Age_{50}+\varepsilon_{ij}$ | $\pi_{0i}=\gamma_{00}+\sum_{k=1}^{7} \gamma_{0k}Z_{ik}+\zeta_{0i}$ $\pi_{1i}=\gamma_{10}+\sum_{k=1}^{8} \gamma_{1k}Z_{ik}+\zeta_{1i}$ | $\begin{matrix} Y_{ij}=\gamma_{00}+\sum_{k=1}^{7} \gamma_{0k}Z_{ik}+\gamma_{10}Age_{50}+\sum_{k=1}^{8} \gamma_{1k}Z_{ik}Age_{50} \\ +(\zeta_{0i}+\zeta_{1i}Age_{50}+\varepsilon_{ij}) \end{matrix}$ |

*Notations*: Y_ij_ is the response variable for each individual “i" and age at visit “j”. $\pi_{0i}$is the level-1 intercept for individual i; $\pi_{1i}$is the level-1 slope for individual i; $\gamma_{00}$is the level-2 intercept of the random intercept $\pi_{0i}$; $\gamma_{10}$is the level-2 intercept of the slope $\pi_{1i}$; $Z_{ik}$is a vector of fixed covariates for each individual i that are used to predict level-1 intercepts and slopes; $\zeta_{0i}$and $\zeta_{1i}$are level-2 disturbances; $\varepsilon_{ij}$is the within-person level-1 disturbance. In model D, an additional $Z_{ik}$ variable is added for Age_50_, to account for quadratic age changes in the fixed effects portion of the model, which increased the number of k terms from 7 to 8 between models C and D.

Model D’s improvement in fit compared to the simpler models was evaluated using Deviance, AIC and BIC statistics as well as pseudo-R^2^. In addition, residuals were plotted against predicted values to assess their normality. It is worth noting that the models were fit using the entire BLSA cohort (n=3005) and not only those who were eligible for the main analysis to improve reliability of predicted estimates. Finally, empirical Bayes estimators of outcomes Y_ij_ were predicted from Model D at specific ages using the following method, after estimating the random effects ($\zeta_{0i}$ for the intercept and $\zeta_{1i}$for the slope) for each individual *i*:

| Intercept | $\pi_{0i}=\gamma_{00}+\sum_{k=1}^{7} \gamma_{0k}Z_{ik}+\zeta_{0i}$ |
| --- | --- |
| Slope | $\pi_{1i}=\gamma_{10}+\sum_{k=1}^{8} \gamma_{1k}Z_{ik}+\zeta_{1i}$ |
| Prediction | $Y_{ij}=\pi_{0i}+\pi_{1i}(Age_{50})_{i}$ where $(Age_{50})_{i}$is assigned individual mean age at follow-up values centered at age 50, thus positive values if Age>50 and negative values if Age<50. |

Y_ij_ in this case is the predicted value of central adiposity for a specific measure j (1=WC, 2=WHR) and individual i. The uppermost quintile per sex was used to define the cutoff point for Elevated Central Adiposity (ECA). Slopes$\pi_{1i}$ were estimated for each adiposity measure j 1=WC, 2=WHR) and individual i, taking into account non-linear changes with age (i.e. the age-square term) at individual-level mean follow-up age and those were interpreted as annual rate of change in each measure between ages 50 y and mean follow-up age. The uppermost quintile per sex was used to define the cutpoint for the binary outcome “Significant Increase in Central Adiposity” or SICA.

**Sources**:

1. Singer JD, Willet JB, eds. Applied Longitudinal Data Analysis: Modeling change and event occurrence. New York: Oxford University Press, 2003.
