# Supplementary figures and images for: Vitamin D receptor and megalin gene polymorphisms are associated with central adiposity status and changes among US adults
Source: J Nutr Sci. 2013 Oct 30;2:e33. doi: 10.1017/jns.2013.19 (PMC4153078; doi:10.1017/jns.2013.19)

## Slide 1
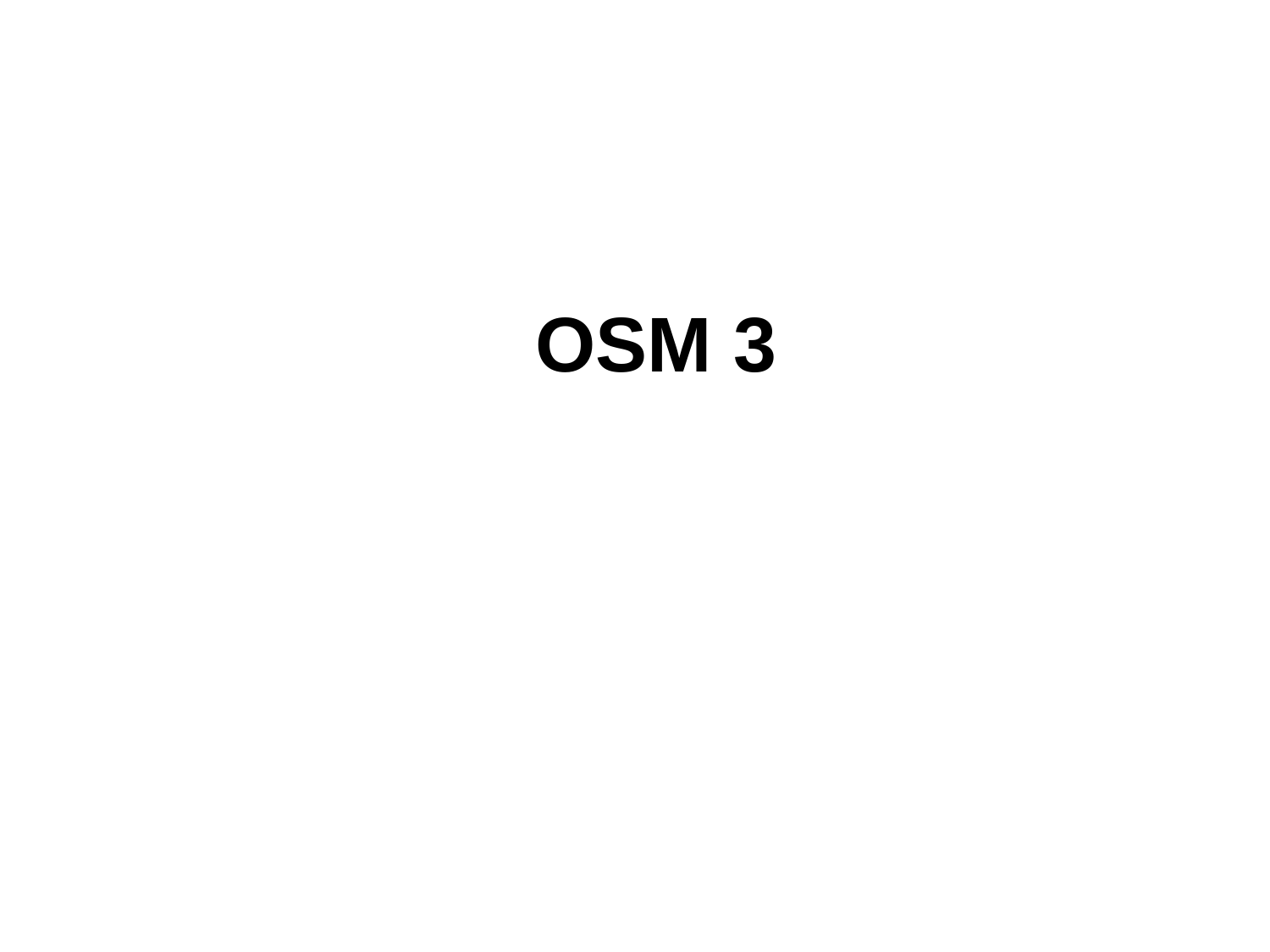

# OSM 3

## Slide 2
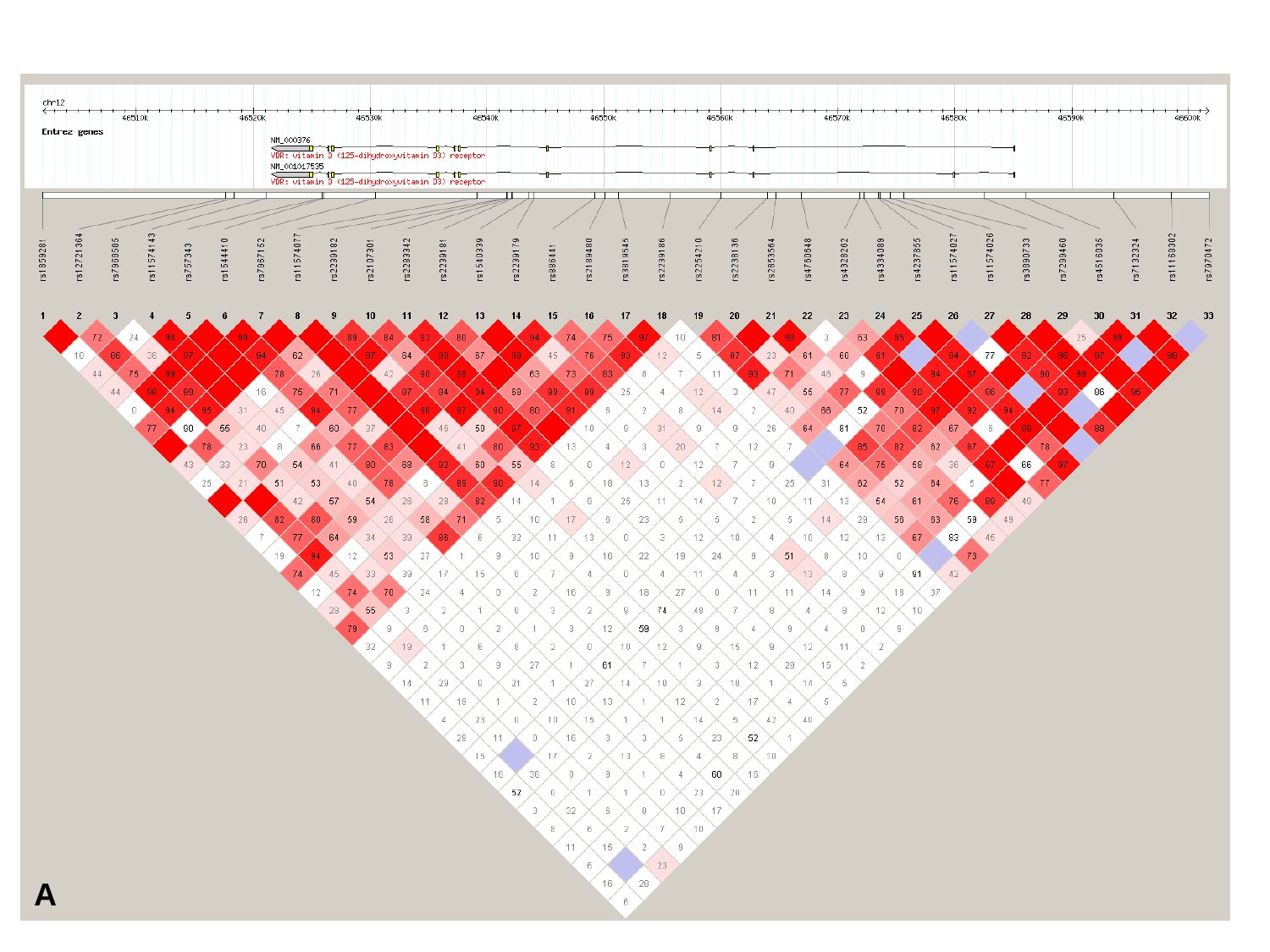

A

## Slide 3
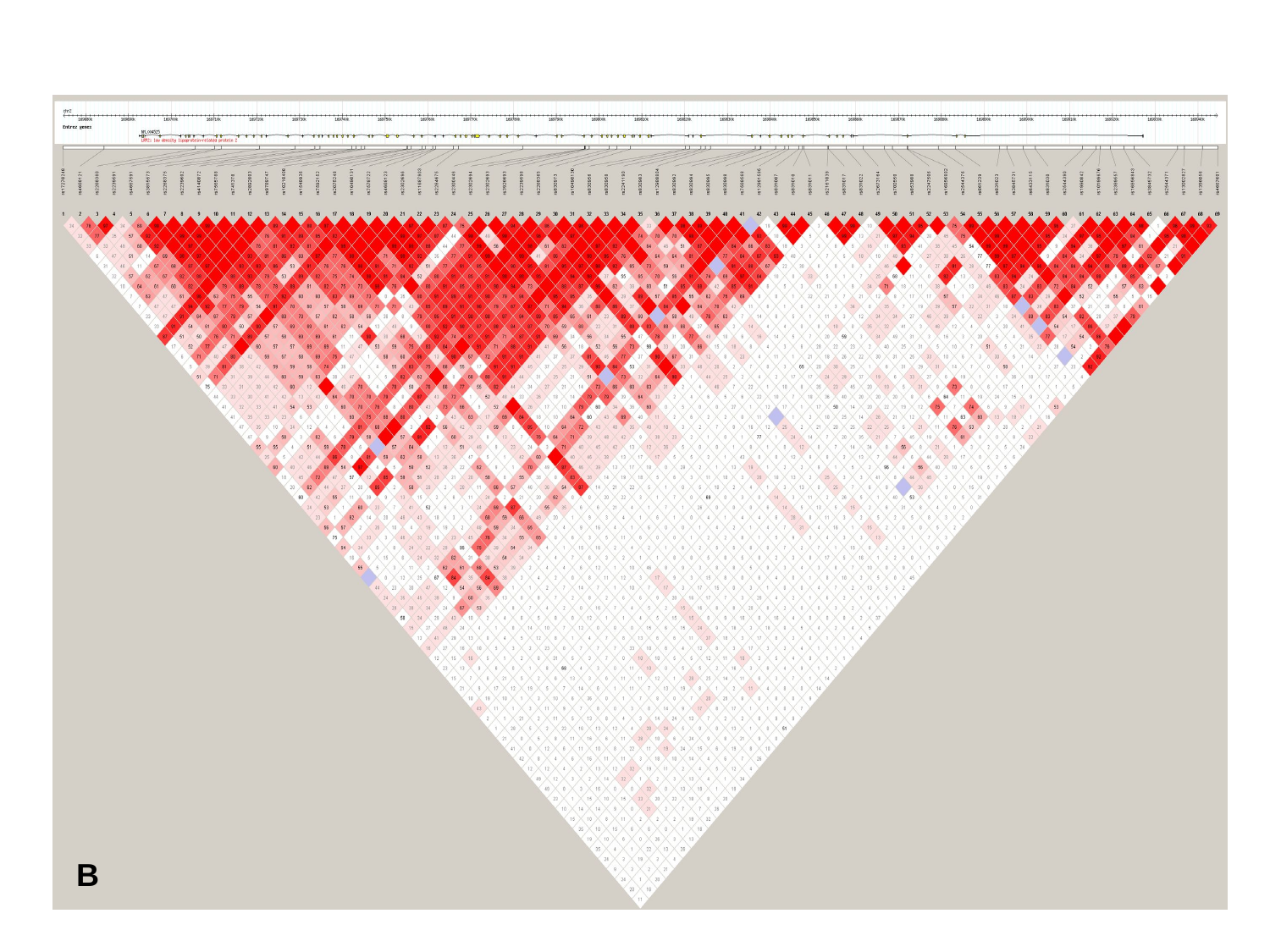

B

Supplement: Supplementary Material — Supplementary information supplied by authors. [file S2048679013000190sup003.ppt]
